# Isoimperatorin therapeutic effect against aluminum induced neurotoxicity in albino mice

**DOI:** 10.3389/fphar.2023.1103940

**Published:** 2023-04-18

**Authors:** Peramaiyan Rajendran, Duaa Althumairy, Mohammad Bani-Ismail, Gamal M. Bekhet, Emad A. Ahmed

**Affiliations:** ^1^ Department of Biological Sciences, College of Science, King Faisal University, Al-Ahsa, Saudi Arabia; ^2^ Centre of Molecular Medicine and Diagnostics, Department of Bio-Chemistry, Saveetha Dental College and Hospitals, Saveetha Institute of Medical and Technical Sciences, Saveetha University, Chennai, Tamil Nadu, India; ^3^ Department of Basic Medical Sciences, Faculty of Medicine, Aqaba Medical Sciences University, Aqaba, Jordan; ^4^ Department of Zoology, Faculty of Science, Alexandria University Egypt, Alexandria, Egypt; ^5^ Laboratory of Molecular Physiology, Zoology Department, Faculty of Science, Assiut University, Assiut, Egypt

**Keywords:** neurotoxicity, ALCL, inflammation, oxidative stress, isoimperatorin

## Abstract

**Background:** Although aluminum (Al) is not biologically crucial to the human body, classical studies have demonstrated that excessive human exposure to Al can induce oxidative damage, neuroinflammatory conditions and neurotoxic manifestations implicated in Alzheimer’s disease (AD). Exposure to Al was reported to be associated with oxidative damage, neuroinflammation, and to enhance progressive multiregional neurodegeneration in animal models. Several plant-derived natural biomolecules have been recently used to reduce the toxic effects of Al through decreasing the oxidative stress and the associated diseases. A good candidate still to be tested is an active natural furanocoumarin, the isoimperatorin (IMP) that can be extracted from Lemon and lime oils and other plants. Here, we examined the neuroprotective effects of IMP on aluminum chloride (AlCl3)-induced neurotoxicity in albino mice.

**Methods:** Twenty-four male albino mice were used in this study. Mice were randomly devided into 5 groups. The first group was given distilled water as a control, the second group was given AlCl3 orally (10 mg/wt/day) starting from the 2nd week to the end of the 6th week, the third group received AlCl3 orally and IMP interperitoneally, i. p. (30 mg/wt/day) starting from week 2 till week 6 where IMP was supplement 1st and then 4 h later AlCl3 was given to mice. The fourth group received the control (IMP 30 mg/wt, i. p.) from the 2nd week till the end of the experiment. Rodent models of central nervous system (CNS) disorders were assessed using object location memory and Y-maze tests in 6th week began. Essential anti-inflammatory and oxidative stress indicators were evaluated, including interleukin-1 β (IL-1β), tumor necrosis factor α (TNF-α), malondialdehyde (MDA), total antioxidant capacity (TAC), and catalase activity (CAT). In addition, serum levels of brain neurotransmitters such as corticosterone, acetylcholine (ACh), dopamine and serotonin in brain homogenates were measured calorimetrically.

**Results:** The study results revealed that the daily treatment of AlCl3 upregulated the TNF-α and IL-1β levels, increased MDA accumulation, and decreased TAC and CAT activity. In addition, aluminum induced a reduction in concentrations of ACh, serotonin and dopamine in the brain. However, IMP significantly ameliorates the effect of AlCl3 through modulating the antioxidant and regulating the inflammatory response through targeting Nrf2 (NF-E2-related factor 2) and mitogen-activated protein kinase (MAPK).

**Conclusion:** Thus, IMP might be a promising treatment option for neurotoxicity and neurodegenerative diseases, such as Alzheimer’s disease and Parkinson’s disease, which are associated with neuro-inflammation and oxidative stress.

## 1 Introduction

Aluminum is one of the most abundant metals found in the earth crust. It is found in many products, making humans prone to daily exposure (Abbas et al., 2022; Hamdan et al., 2022; Turkez et al., 2022). The daily intake of aluminum depends up on the food type. Moreover, some vaccines contain Al salts as adjuvants, which boost the body’s response to the vaccine (Del Giudice et al., 2018; Nouchikian et al., 2018; Principi and Esposito, 2018; Shi et al., 2019; Goullé and Grangeot-Keros, 2020). In addition, drugs used to treat ulcers contain high percentages of Al (Mei and Yao, 1993; Dordevic et al., 2019). Moreover, studies have indicated that antacids contain various Al salts and other metals as active ingredients. Consequently, metal toxicity can be induced by antacid consumption or abuse (Shrivastava et al., 2018; Rahimzadeh et al., 2022). Al affects bone formation and remodeling. Increasing doses of Al slow down osteoblast and osteoclast activity and cause osteomalacia and adynamic bone disease in bone, both clinically and experimentally. Humans and animals’ levels of parathyroid hormone (PTH) are disrupted by Al. In Al-dependent osteotoxicity, altered PTH levels may play a major or even minor role. Al causes a microcytic anemia in hematopoietic tissue, which isn't reversible by iron (Jeffery et al., 1996). However, the blood-brain barrier is relatively highly permeable to Al, leading to its accumulation in the brain. Al accumulation in the brain results in central nervous system (CNS) dysfunction, leading to motor, cognitive and behavioral problems (Wang, 2018; Ishaq et al., 2021; Roe, 2021). On the other hand, patients doing kidney dialysis can experience neurobehavioral symptoms, seizures, CNS toxicity, and even die from Al exposure in the dialysis fluid (Chen et al., 2018; Inan-Eroglu and Ayaz, 2018; Ravat and Gosala, 2018; Lukiw et al., 2019).

In animal models, AlCl3_,_ has been reported to stimulate inflammation, dysfunction in synaptic system and neurodegeneration in several areas of the brain and spinal cord. Mechanistic studies clarified that inflammation stimulates the IκB kinase protein to phosphorylate inhibitor kappa B (NF-кB), allowing the translocation of NF-кB dimers into the nucleus, where they bind to the promoter site of the target gene. This pathway is triggered by certain pro-inflammatory mediators, such as tumor necrosis factor α (TNF-α) and interleukin-1 β (IL-1β). Inflammatory mediators are also regulated by mitogen-activated protein kinases (MAPKs), including c-Jun NH2-terminal kinase (JNK), extracellular signal-regulated kinase (ERK), and p38 in mammals (Zhou et al., 2018; Ji et al., 2021; Zong et al., 2021; Mehrbeheshti et al., 2022). Related to that, the phosphorylation events caused by AlCl3 stimulation can activate JNK, ERK, and p38 (Mehrbeheshti et al., 2022; Tsai et al., 2022). Al exposure and it subsequent accumulation in the brain was shown to be associated with neurodegenerative disorders, such as Parkinson’s disease and Alzheimer’s disease (AD) (Dalla Torre et al., 2019; McLachlan et al., 2019; McLachlan et al., 2020; Bondy and Campbell, 2021; Hassan and Kadry, 2021; Kawahara et al., 2021; Zeng et al., 2021; Abbas et al., 2022). These disorders are characterized by changes in the levels of neurotransmitters, i.e., acetylcholine (Ach). Thus, this promotes neuroinflammation and neurodegeneration, resulting in Alzheimer’s disease. These toxic effects stimulate complex mechanisms to defend against toxicity, such as heme oxygenase 1 (HO-1), NAD(P)H: quinone oxidoreductase 1 (NQO-1), superoxide dismutase 1, glutathione peroxidase, thioredoxins, and glutathione S-transferase (Li et al., 2014), which are activated by NF-E2-related factor 2 (Nrf2) in the CNS (Loboda et al., 2016; Wang et al., 2019). In addition, HO-1 and NQO-1 were demonstrated to reduce oxidative stress and maintain mitochondrial integrity, thereby protecting neurons (Yang et al., 2015). In parallel with that, patients with dementia display altered levels of HO-1 and NQO-1 expression (Wang et al., 2000). However, overexpression of Nrf2 was found to provide sufficient protection against neurotoxicity (Wang et al., 2019). Thus, to reduce symptoms of oxidative stress and treating neurodegenerative diseases, stimulating the Nrf2 signaling pathway might be a valuable tool.

Several plant-derived antioxidants that protect against metal toxicity, inflammation and apoptosis-related neurodegenerative disorders have recently been identified (Cherrak et al., 2016; Yadav et al., 2016; Lakey-Beitia et al., 2021). Angelica dahurica, Rhizoma et Radix Notopterygii, and Radix Peucedani contain isoimperatorin (IMP), an active natural furanocoumarin (Lai et al., 2021). Lemon and lime oils also contain IMP. Among its pharmacological effects, IMP has anti-hypertensive (Hwang et al., 2017), anti-inflammatory (Wijerathne et al., 2017; Chen et al., 2021), anti-tumor (Yang et al., 2018a), antibacterial (de Souza et al., 2005; Pokharel et al., 2006), and liver protective (Pokharel et al., 2006) effects, including diastolic vasoactivity (Lai et al., 2021), among other effects (Wijerathne et al., 2017; Kiyonga et al., 2021; Lai et al., 2021; Chahardoli et al., 2022; Kim et al., 2022; Liu et al., 2022). Dietary furocoumarin IMP was shown to stimulate glucagon-like peptide secretion and reduces blood sugar in rats by activating the G protein-coupled bile acid receptor (Wang et al., 2017). In addition, IMP was found to enhance adipocyte differentiation in lipodystrophic patients. These patients had a triglyceride storage defects and severe insulin resistance because of ectopic lipids accumulate in adipose tissue. As a result of increased fat storage capacity in adipose tissue with low-fat mobilization, fat mass expands which is the most optimal method of storing lipids (Jiang et al., 2019). This study tested whether IMP protects albino mice against AlCl3-induced behavioral deficits, neurotransmitter abnormalities, and oxidative inflammatory damage.

## 2 Materials and methods

### 2.1 Chemicals

Sigma-Aldrich Co., (St. Louis, MO, United States) provided IMP and AlCl3. Stock solutions were prepared every week. The AlCl3 was dissolved in distilled water, while the IMP was dissolved in DMSO first, then diluted in normal saline to 0.1% DMSO. Aseptic conditions were used to prepare and inject the drugs.

### 2.2 Animal groups

Twenty-four male albino mice were used in this study. Each group included six mice (6–8 week old). Throughout the research, all methods were conducted according to relevant guidelines and regulations at King Faisal University. In a normal laboratory atmosphere, mice were kept at a temperature of 24°C and were subjected to a 12/12-h light/dark cycle. Mice were randomly divided into 5 groups. The first group was used as a control an received distilled water, the second group was given AlCl3 orally (10 mg/wt/day) starting from the 2nd week to the end of the 6th week, the third group received AlCl3 orally and IMP interperitoneally, i. p. (30 mg/wt/day) starting from week 2 till week 6 where IMP was supplement 1st and then 4 h later AlCl3 was given to mice. The fourth group received the control (IMP 30 mg/wt, i. p.) from the 2nd week till the end of the experiment. At the end of the experiment, mice were sedated with ether before being culled. To weigh the brain after excision, we used a Nimbus balance (MK, UK). In accordance with the protocol, the biochemical parameters were measured immediately after the brains were homogenized.

### 2.3 Object-location memory

6 weeks after beginning, an object recognition test was conducted. We put green and yellow color small plates Each mouse was adapted for 10 min in a 120 × 80 cm box. Intbox1 30 min’ intervals, they started training. 5 min were given to each mouse to practice object-location memory in a quadratic box (120 × 80 cm, 25 cm high). The apparatus was set up with two identical plastic objects 120 cm from the wall. There was a 1-day break between training and testing. The mice were tested with two identical objects 120 cm from the wall. Time spent looking at objects was used to measure animals’ behavior.

### 2.4 Y-Maze

6 weeks after beginning, an Y-Maze test was conducted. Studies have shown that excessive spontaneous alteration behavior is linked to enhanced cognitive performance. We followed Buried Food Test, the protocol have published by Mu Yang and Dr. Jacqueline N. Crawley. Simple Behavioral Assessment of Mouse Olfaction (Yang and Crawley, 2009). A Y-maze was used to analyze mice’s behavior. It was made of brown pointed sheets with three arms that were 60 cm long, 15 cm high, and 15 cm wide at the bottom and top. Mouse sessions lasted from three to 5 min. In the right arms of Y, there is food. The mouse capability to remember if food is located in one arm or the other (Limnios et al., 2022). By this way conditions could affect learning and memory and help to analyze the mice behavior. For the food identification, each mouse’s arm position was recorded manually 3 times in different days for 5 min each.

### 2.5 Evaluation of brain neurotransmitters

The acetylcholine levels were measured in brain supernatants using a colorimetric assay kit (BioVision Inc., Waltham, United States) at 570 nm (Contoli et al., 2017). Colorimetric measurements of serotonin (Chellammal et al., 2019) and dopamine (Chellammal et al., 2019) were also conducted (BioVision).

### 2.6 Quantification of markers of oxidative damage in brain tissue

A competitive ELISA kit (Cell Biolabs, San Diego, CA, United States) was used to measure malondialdehyde (MDA). ELISA immunoassay for MDA-protein adduct products was used for this assay. Moreover, total capacity of antioxidants was measured Capacity Assay Kit (Cell Biolabs) based on copper (II) being reduced to copper (I) by antioxidants. By reducing copper (I) ions, a coupling chromogen increases 490 nm absorbance. The net absorbance was compared to that of the standard curve based on known uric acid concentrations. An ELISA kit for mice catalase (CAT) (Cusabio, Wuhan, China) was used to measure catalase activity.

### 2.7 Western blot

Western blot was conducted according to a standard method. Isolated proteins were electrophoresed on SDS-PAGE gels and then transferred to PVDF membranes. The blots were probed overnight at 4°C using primary antibodies (Supplementary Table S1) after being blocked with 5% nonfat dry milk for 60 min. Blots were then incubated with the suitable HRP-conjugated secondary antibody (Supplementary Table S1) for 60 min at room temperature. To visualize the protein bands, we used Digit Blot (a 3,600-00-C-) Scanner. The untreated control band was normalized to 1 with Image Studio Lite software (Lincoln, NE, United States). We repeated each experiment three times.

### 2.8 Estimation of pro-inflammatory biomarkers

E-labscience (Beijing, China) ELISA Kits were used to determine IL-1β and TNF-α levels in the brain. The Sandwich-ELISA principle is used in this ELISA kit. A TNF-α and IL-1β specific antibody has been pre-coated on the micro ELISA plate in this kit. ELISA plates are filled with standards or samples and specific antibodies are added. A biotinylated detection antibody and Avidin-Horseradish Peroxidase (HRP) conjugate are added successively to each microplate well and incubated. Components that are not needed are washed away. The substrate solution is added to each well. Wells containing Mouse TNF-α and IL-1β, biotinylated detection antibody, and Avidin-HRP conjugate will be blue. When stop solution is added, the enzyme-substrate reaction stops and the color turns yellow. In spectrophotometry, optical density is measured at 450 nm ± 2 nm. A high OD value means more Mouse TNF-α and IL-1β. Using the standard curve, we can calculate the concentration of Mouse TNF in the samples. A Molecular Devices SpectraMaxTM Multimodal plate reader was used to get all the readings.

### 2.9 Histopathology

The brain tissues were embedded in paraffin and fixed in 10% formalin to prepare them for histopathology. Four-mm-wide sections of embedding brain tissues lobes stained with H&E. An optical microscope was used to study the prepared sections. A Leica D6000 microscope (Leica, Wetzlar, Germany) was used for measurements, and microscopy obtained images from cerebral cortex (×200 magnification).

### 2.10 Statistical analysis

Data analysis for behavioral, biochemical and histopathological evaluations was done using Prism 8 (GraphPad). Differences between groups were analyzed using ANOVA, followed by Tukey’s *post hoc* test. Data are presented as means ± SD. For in viv*o* experiments, at least three independent experiments were performed. The probability value of 0.05 was considered to be significant variation.

## 3 Results

### 3.1 Effect of IMP on AlCl3-induced body weight of experimental animals

**FIGURE 1:**
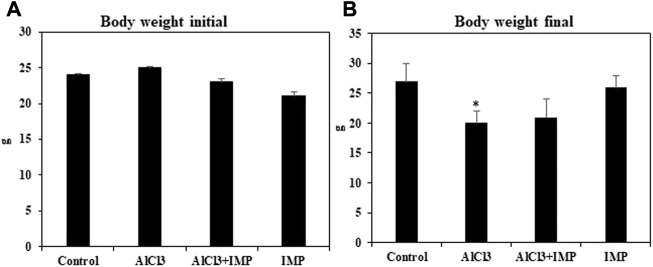
Effect of IMP on body and brain weight of experimental animals. **(A)** Body weight of animal starting date (g). **(B)** Body weight of the animal at the end of experimental date (g). The difference between the treatment and control groups was statistically significant; *p < 0.05, #*p* < 0.05 was considered statistically significant. Values are expressed as mean ± SD, *n* = 3. *p < 0.05 indicates significant differences between the AlCl3 alone group and the control group. #*p* < 0.05 shows significant differences between AlCl3 alone and IMP with AlCl3.

First, we have compared the body weight (initial and end) of treated groups. According to the statistical analysis the end of body weight values show significant differences among the studied groups, (Figures 1A, B).

### 3.2 Effect of IMP on mice behavior

#### 3.2.1 Object-location memory test

**FIGURE 2:**
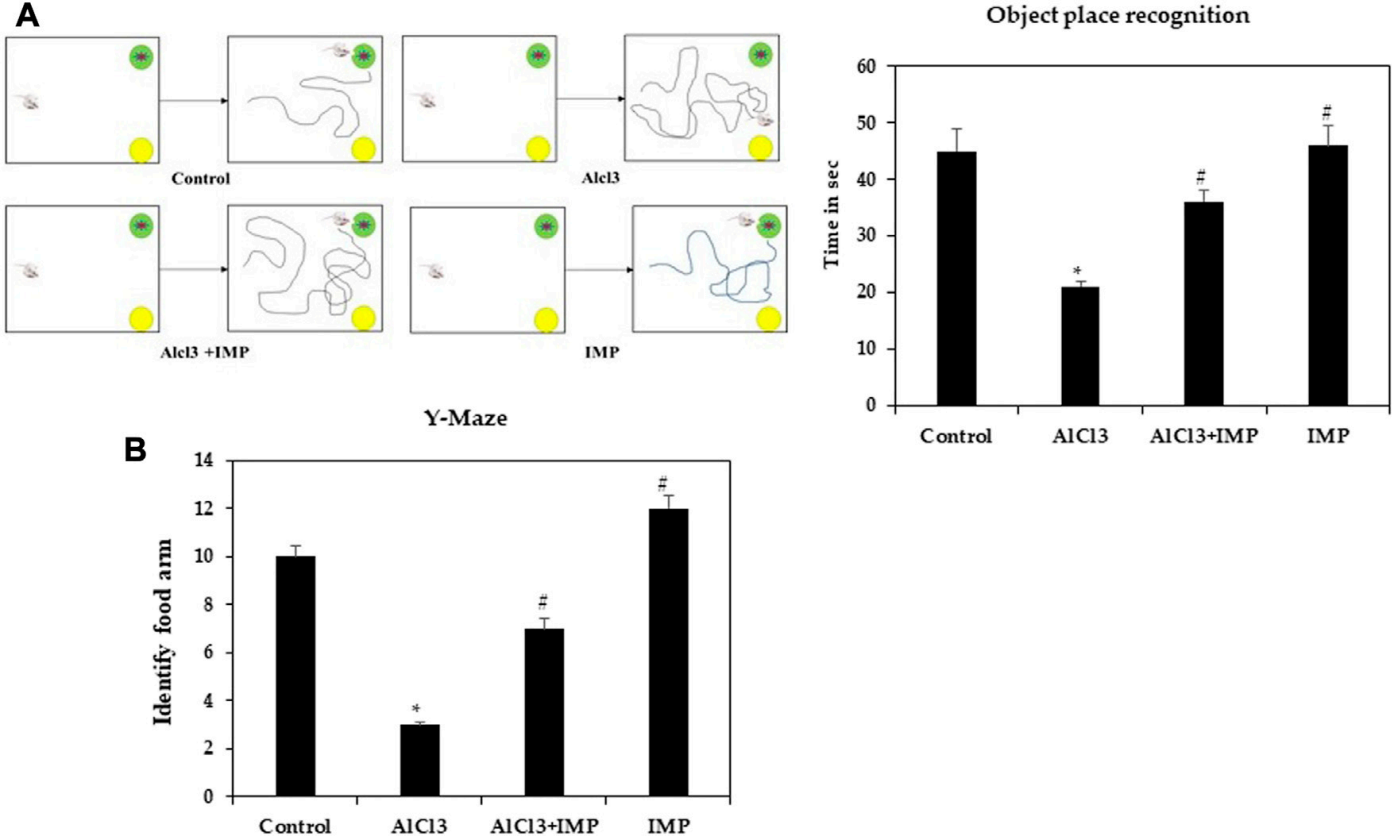
Spatial memory learning of mice. **(A)** Object place recognition test. **(B)** Y-maze test. The difference between the treatment and control groups was statistically significant; *p < 0.05, #*p* < 0.05 was considered statistically significant. Values are expressed as mean ± SD, *n* = 3. *p < 0.05 indicates that there are significant differences between the AlCl3 alone group and the control group. #*p* < 0.05 shows significant differences between AlCl3 alone and IMP with AlCl3.

In rodent models of CNS disorders, object-location memory is used to assess cognition, and specifically spatial memory and discrimination. The effect of IMP treatment on novel object memory was also explored. A noticeable increase in the object memory index was found in the control group when the novel object-location memory test was compared to the training session. Treatment with AlCl3 also worsened amnesia (Figure 2A). However, pretreatment with IMP (30 mg/kg) prevented deficits in memory (*p* 0.05) caused by long-term exposure to AlCl3 (Supplementary Vedio S1). Therefore, IMP protects mice from the effects of AlCl3 on memory and object location.

#### 3.2.2 IMP prevented memory damage

Spatial working memory was analyzed using a Y-maze task. Food rewards were placed around the arms of mice treated with AlCl3 were less likely to enter. When mice received IMP alone or in combination with AlCl3, altered behavior was significantly modulated (*p* < 0.05), indicating that IMP improved memory in AlCl3-treated mice (Figure 2B). There were no significant differences between group IV (Figure 2B) and the control group in response to the food rewards placed in the arms [(Supplementary Vedio S1)]. Then, IMP may improve the working memory.

### 3.3 IMP improved ach levels in mice with AlCl3-induced neurotoxicity

**FIGURE 3:**
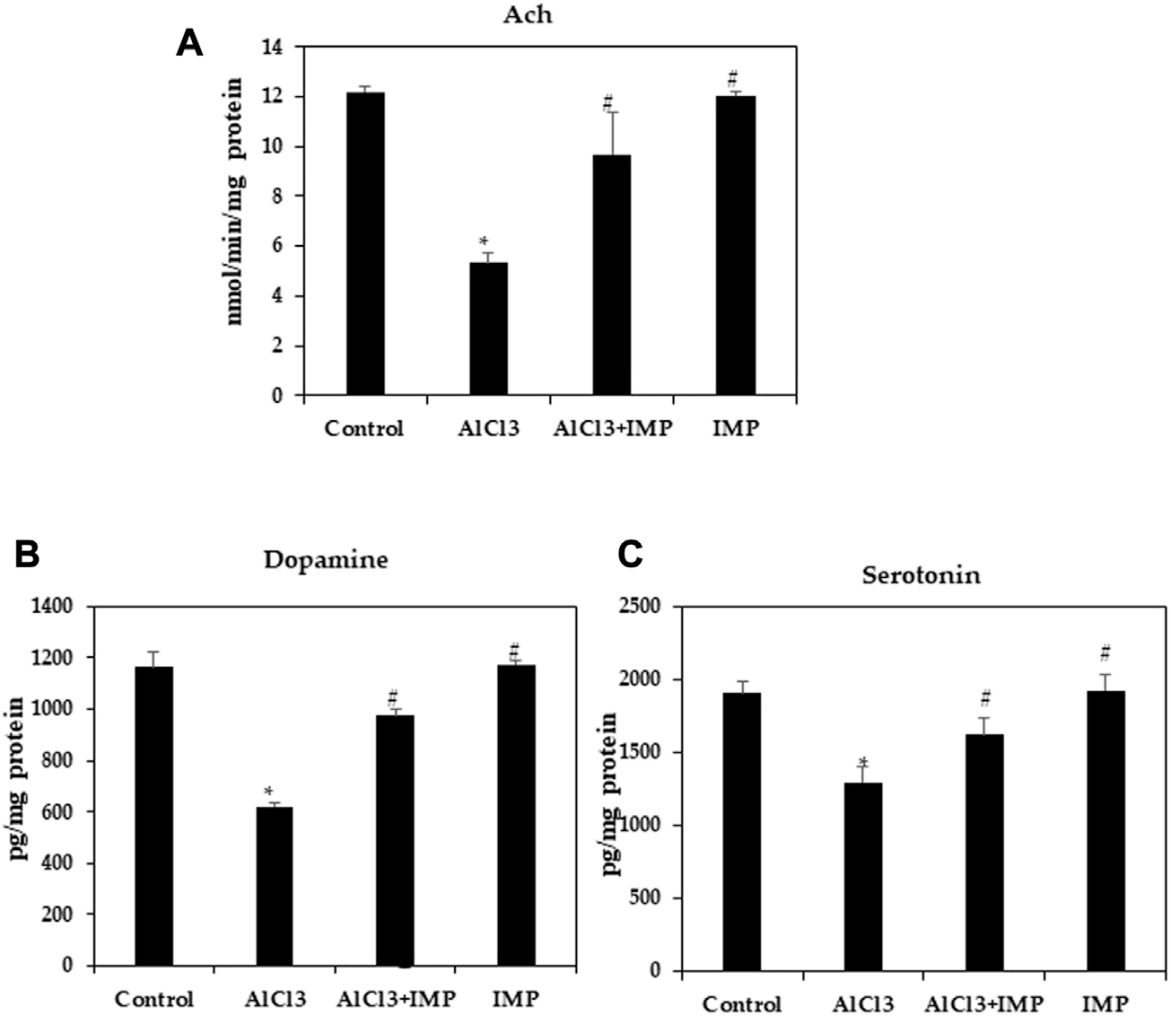
Effect of IMP on the level of Ach, Serotonin and Dopamine. **(A)** Level of acetylcholine concentration (nmol/min/mg protein). **(B)** Level of dopamine (pg/mg protein). **(C)** Level of serotonin (pg/mg protein). The difference between the treatment and control groups was statistically significant; *p < 0.05, #*p* < 0.05 was considered statistically significant. Values are expressed as mean ± SD, *n* = 3. *p < 0.05 indicates that there are significant differences between the AlCl3 alone group and the control group. #*p* < 0.05 shows significant differences between AlCl3 alone and IMP with AlCl3.

The acetylcholine, the first identified neurotransmitter, plays a significant role in hippocampal memory and AD pathogenesis. It is synthesized by choline acetyltransferase, AchE, an enzyme found in cerebrospinal fluid and plasma. The AchE is an enzyme that metabolizes Ach at synaptic clefts (Karami et al., 2021; Huang et al., 2022). To further test neuroprotective effects of IMP on brain damage, we examined cholinergic dysfunction, as indicated by a decreased Ach content (5.5 fold). Figure 3A shows that IMP reversed the reduction in Ach content (5 fold). The AlCl3 group had significantly lower dopamine and serotonin levels than the control group, and the AlCl3 + IMP group had significantly higher levels than the AlCl3 group (Figures 3B, C). Our findings suggest that IMP (30 mg/wt) helps alleviate AlCl3-induced neurotoxicity.

### 3.4 IMP downregulates pro-inflammatory markers

**FIGURE 4:**
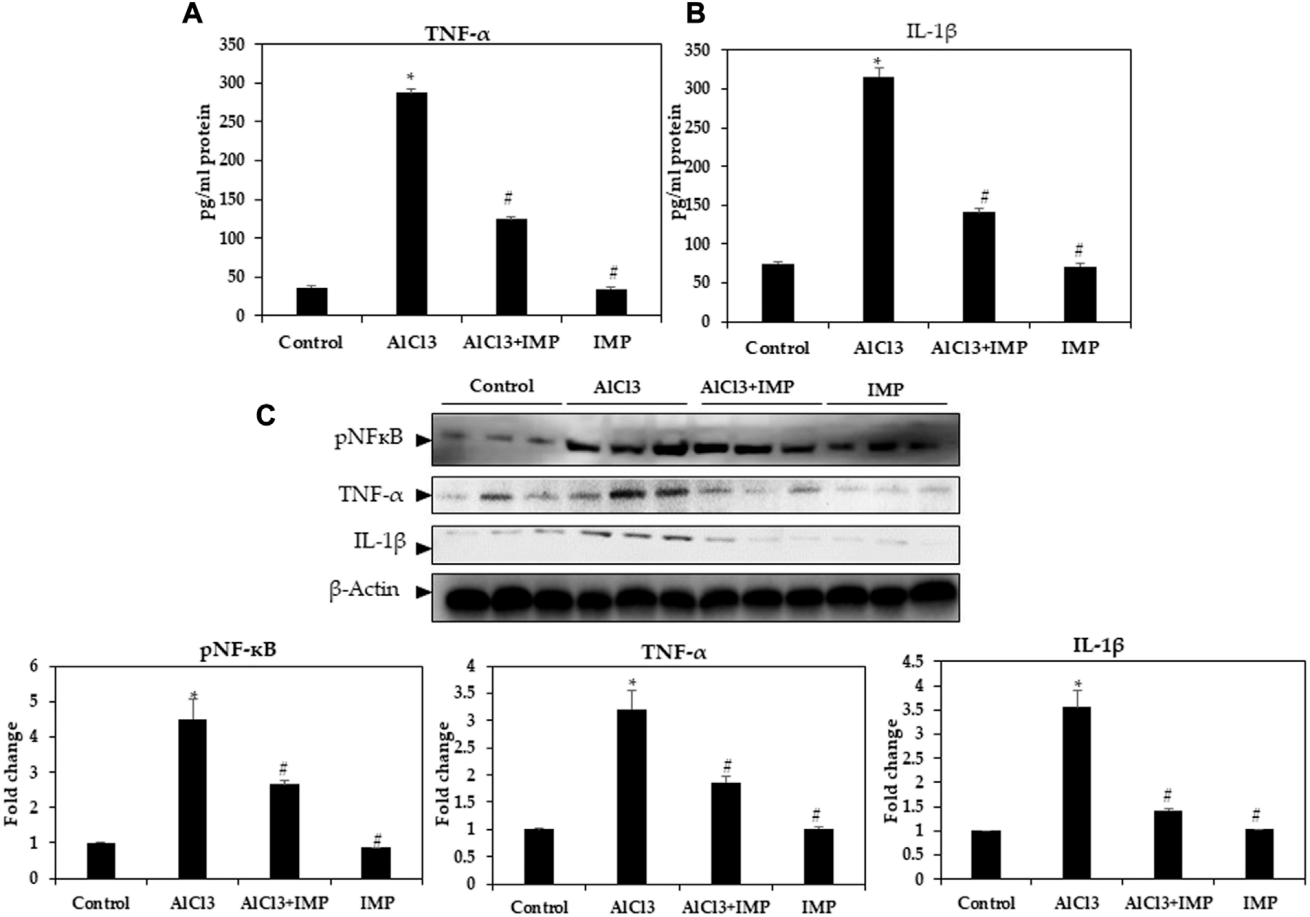
Level of pro-inflammatory cytokines in brain homogenates. **(A)** Level of TNF-α (pg/mg protein). **(B)** Level of IL-1β (pg/mg protein). **(C)** Western blot analysis of NF-кB and the pro-inflammatory cytokine (TNF-α and IL-1β) expression in brain homogenates. The difference between the treated groups and the control group was calculated; *p < 0.05, #*p* < 0.05 was considered statistically significant. Values are expressed as mean ± SD, *n* = 3. *p < 0.05 indicates significant differences between AlCl3 treated group and the control group. #*p* < 0.05 shows significant differences between AlCl3 alone and IMP with AlCl3.

Neuroinflammation and monoamine neurotransmitters are closely related to AlCl3-induced neuronal damage (Stephenson et al., 2018; Ji et al., 2021). Enzyme-linked immunoassay was used to detect pro-inflammatory cytokine levels in mice. In Figures 4A, B, TNF-α and IL-1β levels in mouse brains were remarkably higher after AlCl3 treatment, but IMP (30 mg/wt) induced a significant decrease in these levels. Then, IMP reduced the inflammation caused by AlCl3.

### 3.5 IMP inhibits NF-кB activation and its associated inflammatory cytokines in AlCl3-induced mice

Innate and adaptive immune functions are controlled by NFκB (Mulero et al., 2019; Capece et al., 2022). It also mediates inflammation (Rajendran et al., 2018; Nichols et al., 2019; Peng et al., 2019). In IMP-pretreated and AlCl3-stimulated mouse brain tissues, we tested the efficacy of NFB in suppressing inflammation. Figure 3C illustrates that nuclear protein extracts from the brain tissue showed a remarkable increase in p65 levels after AlCl3 stimulation. AlCl3-induced p65 levels were significantly attenuated by IMP pretreatment. Next, we tested the effect of IMP on TNF-α and IL-1β by Western blotting. The Western blot results showed that AlCl3 stimulation overexpressed TNF-α and IL-1β. However, IMP suppressed this effect significantly, suggesting that IMP suppressed the activation of p65, which further suppressed the expression of inflammatory enzymes and cytokines (Figure 4C).

### 3.6 Effect of aluminum treatment and IMP on LPO and antioxidant enzymes

**FIGURE 5:**
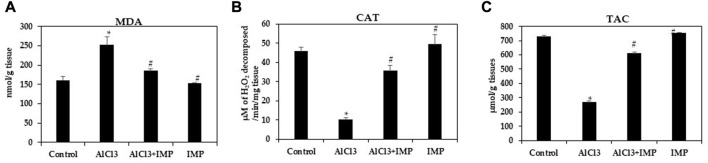
Activity of antioxidant enzymes. **(A)** Formation of MDA in brain homogenates (nmol/g tissue). **(B)** The activity of catalase (µM of H_2_O_2_ decomposed/min/mg tissue). **(C)** Total antioxidant capacity (µmol/g tissues). The difference between the treatment and control groups was statistically significant; *p < 0.05, #*p* < 0.05 was considered statistically significant. Values are expressed as mean ± SD, *n* = 3. *p < 0.05 indicates that there are significant differences between the AlCl3 alone group and the control group. #*p* < 0.05 shows significant differences between AlCl3 alone and IMP with AlCl3.

Various oxidant and antioxidant enzymes tightly regulate ROS production in cells as by-products of oxidative metabolism (Rajendran et al., 2014; Snezhkina et al., 2019). MDA is an indicator of oxidative stress caused by lipid peroxidation in cells and tissue (Fauziah et al., 2018; Subramanyam et al., 2018). Our first experiment was conducted to observe how IMP affected AlCl3-induced MDA levels. MDA levels significantly increased in mice exposed to 10 mg/kg AlCl3 for 6 weeks (Figure 5A). A consistent, significant decrease in Catalase and TAC content was caused by AlCl3 (Figures 5B, C). All of these pro-oxidative stress effects were potently inhibited by the co-administration of IMP. These results suggest that IMP protects the brain from oxidative damage caused by AlCl3.

### 3.7 MAPK signaling pathway is affected by IMP

**FIGURE 6:**
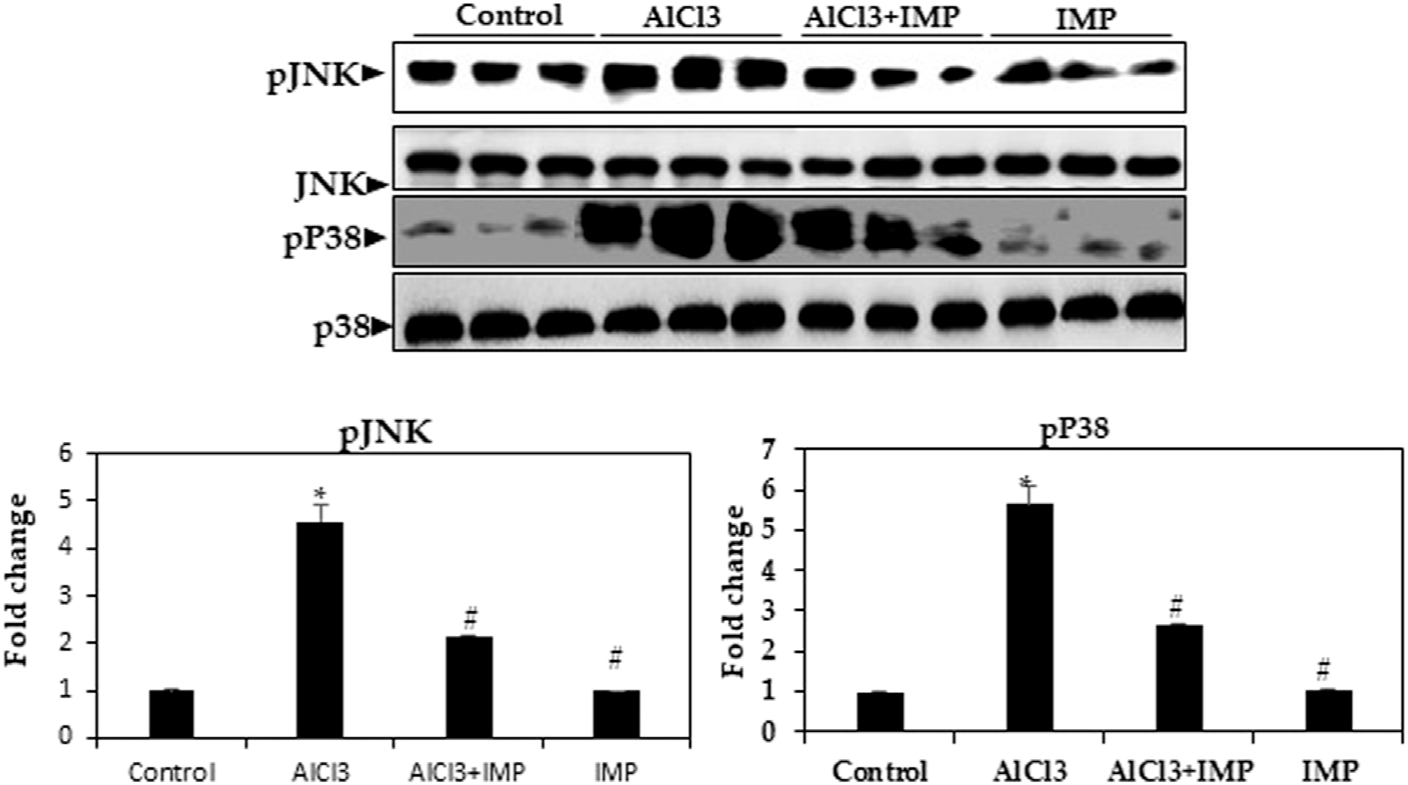
Expression of MAPK kinase in brain homogenates. Western blot analysis of brain homogenates determines the expression of pJNK and pP38—total JNK and total p38 as a loading control. The difference between the treatment and control groups was statistically significant; *p < 0.05, #*p* < 0.05 was considered statistically significant. Values are expressed as mean ± SD, *n* = 3. *p < 0.05 means that there are significant differences between the AlCl3-alone group and the control group. #*p* < 0.05 shows significant differences between AlCl3 alone and IMP with AlCl3.

Inflammation and apoptosis are triggered by MAPKs, including JNK and p38 kinases (Kasuya et al., 2018; Aluko et al., 2021; Ijomone et al., 2021). As shown in Figure 6, AlCl3 increased the phosphorylation of the MAPKs p38 and JNK by AlCl3, while phosphorylation was inhibited by IMP treatment. IMP suppresses p38 and JNK activation. Thus, IMP attenuates inflammation by downregulating MAPK signaling through the inhibition of p38 and JNK activation in AlCl3-induced brain damage in mice.

### 3.8 AlCl3-stimulated ROS generation is attenuated by IMP

**FIGURE 7:**
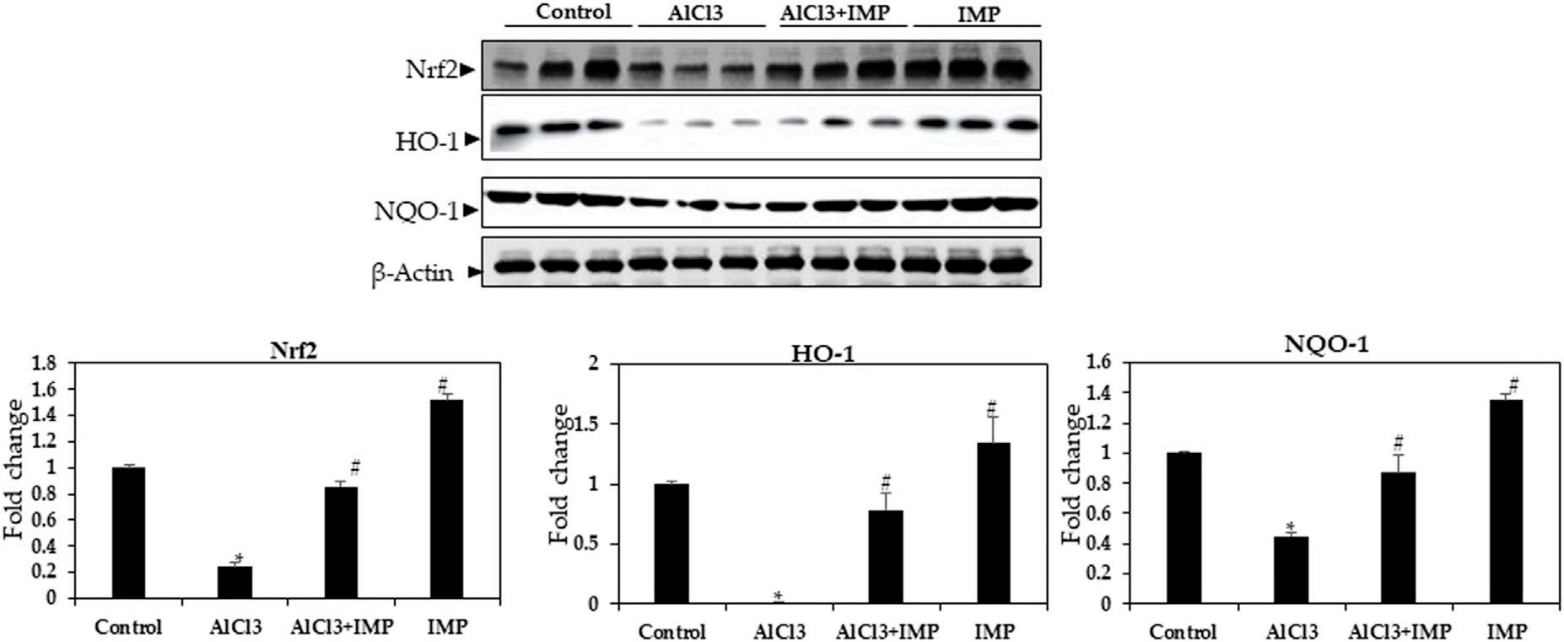
Expression of Nrf2 in brain homogenates. Western blot analysis of Nrf2, HO-1, and NQO-1 expression in control and experimental animals. The difference between the treatment and control groups was statistically significant; *p < 0.05, #*p* < 0.05 was considered statistically significant. Values are expressed as mean ± SD, *n* = 3. *p < 0.05 indicates significant differences between the AlCl3-alone group and the control group. #*p* < 0.05 shows significant differences between AlCl3 alone and IMP with AlCl3.

HO-1 and NQO are antioxidant enzymes that are regulated by the Nrf2 pathway (Wang et al., 2000; Li et al., 2014; Yang et al., 2018b). To understand the molecular mechanisms underlying the protective effects of IMP, we studied the Nrf2 pathway in mouse brain tissue. In mouse brains, exposure to AlCl3 for 6 weeks inhibited Nrf2 expression, which was profoundly restored by the co-administration of IMP (30 mg/wt) (Figure 7). We also evaluated Nrf2-dependent anti-oxidative and detoxifying enzymes. Compared with the AlCl3 group, the AlCl3 + IMP group demonstrated increased protein expression of HO-1 (Figure 6). NQO1 expression was partially rescued by IMP when AlCl3 decreased it. Overall, IMP treatment rescued AlCl3-induced oxidative damage by activating Nrf2.

### 3.9 Histopathological evaluation of brain tissue

**FIGURE 8:**
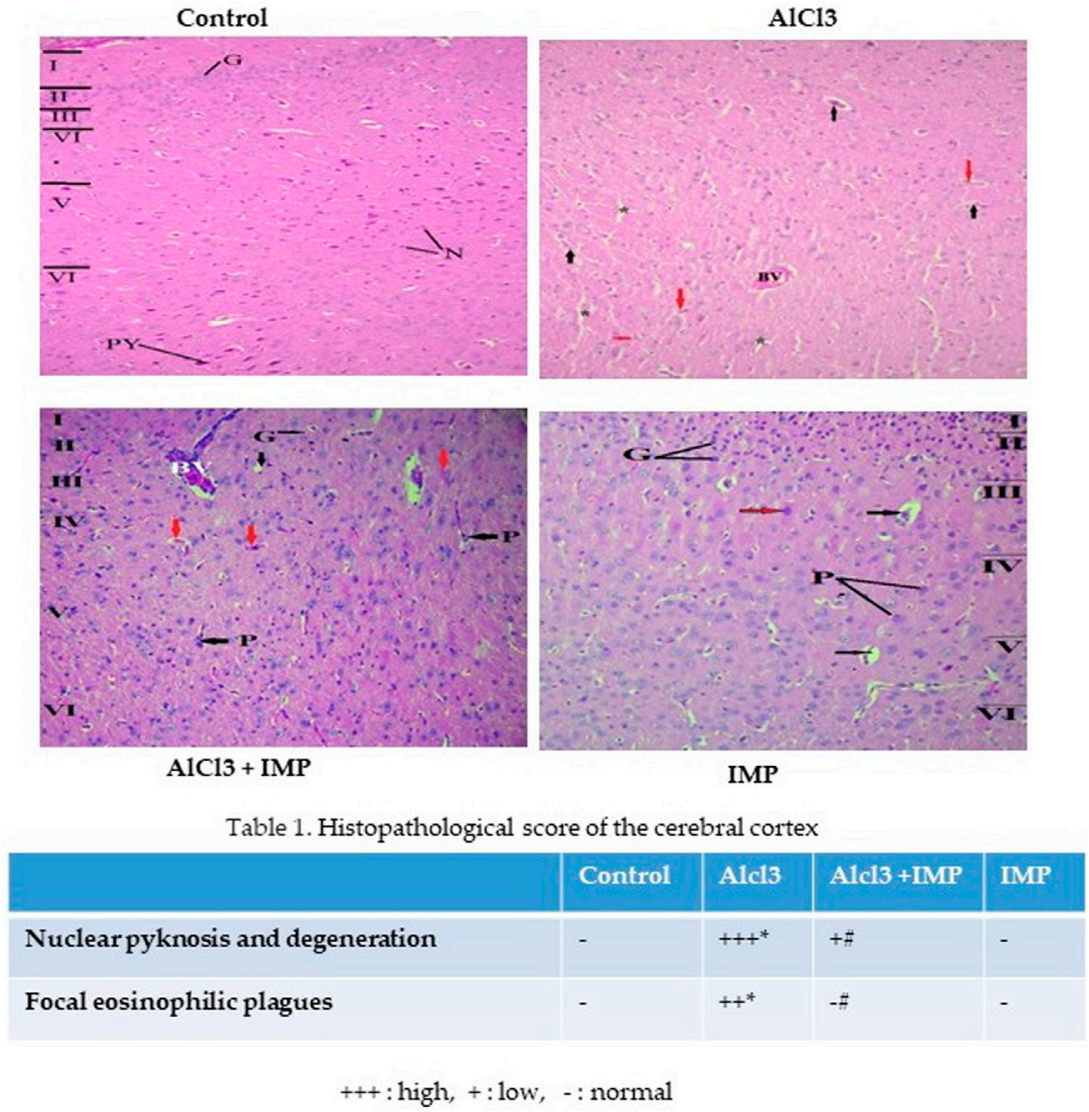
Histopathological analysis of brain tissue. The difference between the treatment and control groups was statistically significant; *p < 0.05, #*p* < 0.05 was considered statistically significant. Values are expressed as mean ± SD, *n* = 3. *p < 0.05 indicates significant differences between the AlCl3-alone group and the control group. #*p* < 0.05 shows significant differences between AlCl3 alone and IMP with AlCl3.

Figure 8 showed cerebral histopathological analysis. Control group showing normal cerebral cortex composed of six layers (I-VI). I- Molecular layer, II- External granular layer, III- External pyramidal layer, IV- Internal granular layer, V- Internal pyramidal layer, VI- Multiform layer. Pyramidal cells (P) have multipolar shape, vesicular nuclei, and basophilic cytoplasm while granular cells (G) have small nuclei and little cytoplasm. Smaller neuroglia cells appear scattered (N). AlCl3 group showing distortion and disappearance of normally arranged cortical layers and congested blood vessels (BV), neurophil vacuolation (black arrows), degenerating neurons with pyknotic nuclei in panel (red arrows) and vacuolation (*). AlCl3 + IMP Showing some disorganized layers, shrunken pyramidal cells (P black arrows) with loss of processes, dark cytoplasm and small darkly stained nucleus while granular cells (G) are surrounded by halos, degenerating neurons with pyknotic nuclei and dilated blood vessel (BV). IMP alone treated animals showing an improvement in histoarchitecture of organized layers, Pyramidal cells (P) and granular cells (G) appear more normal and few of degenerating neurons with pyknotic nuclei (red arrows). Together these data indicate that IMP partially reversed the effects of chronic AlCl3.

## 3 Discussion

In the present study, we show that IMP protects the brain from alunimium toxicity through modulating the levels of antioxidant and anti-inflammatory markers. In addition, it can partially reduce the effects of chronic AlCl3 exposure on object recognition index and spatial working memory.

Neurodegenerative disease progression has been extensively shown to be influenced by oxidative stress, inflammation, and apoptosis (Behl et al., 2021; Merelli et al., 2021; Zarneshan et al., 2022). In this regard, previous studies indicated that Al exposure can induce various neurological disorders (Aly et al., 2011; Hamdan et al., 2022; Turkez et al., 2022). Therefore, chronic exposure to Al in mouse models has frequently been used as an experimental model of neurodegenerative diseases. Supplemented AlCl3 is able pass through the blood-brain barriers and then induces neuroinflammation (Baydar et al., 2003; He et al., 2022). Moreover, AlCl3 is found to activate oxidative mediators (Abbas et al., 2022). Acute and chronic neurodegenerative diseases are closely related to excessive AlCl3 accumulation because they activate ROS production and oxidative neurotoxicity (Khalid et al., 2020; Li et al., 2020; Madi et al., 2020).

The current study results showed that IMP partially reversed the effects of chronic AlCl3 exposure on object recognition index and spatial working memory. During depression, the central serotonergic system is hypofunctional and the brain serotonin levels are lower than normal (Hashmi et al., 2018; Kaur et al., 2022). Consistent with that, AlCl3 reduced brain serotonin levels, while AlCl3/IMP combined treatment maintained adequate levels, which may explain the mitigation of depressive symptoms. In addition, exposure to AlCl3 reduced the ACh concentration in the brain. Suppressed cholinergic transmission was reported to activate the inflammatory system by increasing N-methyl-D-aspartate receptor expression, disrupting cognition, and increasing neurotoxicity (Haider et al., 2020; Amanzadeh Jajin et al., 2021; Firdaus et al., 2022). AlCl3 may cause neurodegeneration through multiple interlocking pathogenic pathways (Wang et al., 2019; Shunan et al., 2021; Hamdan et al., 2022). However, one of the reasons that IMP is effective against Al-induced neurotoxicity is probably due to its anti-inflammatory action on cytokines and its role in normalizing ACh levels in the brain.

Activation of microglia in brain disease was reported to be associated with the release of copious quantities of pro-inflammatory cytokines, which were believed to contribute to neuronal death and degeneration during neuroinflammation and brain disease (Ahmad et al., 2019). In the Nrf2/HO-1 axis of the AlCl3 group, NF-кb expression showed a significant increase, thereby increasing pro-inflammatory cytokines, including TNF-α and IL-1β, causing neuroinflammation in the brain (Hamdan et al., 2022). Natural flavonoids have been studied for their multiple neuroprotective properties, including their role in suppressing inflammation and neuronal apoptosis as well as in boosting neuronal survival and memory (Infante-Garcia and Garcia-Alloza, 2019). Our findings indicate that IMP suppressed the pro-inflammatory mediators induced by AlCl3 and consequently attenuated AlCl3-induced neuroinflammation in mice. MAPK pathway proteins such as p38, JNK, and ERK1/2 have been reported to contribute to NF-κB activation. The phosphorylation of MAPKs is essential for regulating cell division, proliferation, and differentiation. Ruther more, MAPKs can trigger apoptosis (Kahkhaie et al., 2019; Yu et al., 2019; Ijomone et al., 2021; Huang et al., 2022). Moreover, as shown above, IMP suppressed the activation of p38 and JNK caused by AlCl3. Based on that, IMP appers to exert anti-inflammatory effects in mice treated with AlCl3 through inhibiting the MAPK signaling pathway.

Neurodegenerative diseases are associated with an imbalance between free radicals (i.e., ROS) and antioxidants and antioxidant enzymes (Ajayi et al., 2021; Bardallo et al., 2022; Kısadere et al., 2022), leading to the induction of oxidative stress (Haider et al., 2020; Amanzadeh Jajin et al., 2021; Abdel-Salam, 2022; Mishra et al., 2022). The main antioxidant enzymes that protect against the harmful effects of ROS in cells are SOD and CAT, are among the brain’s most prominent antioxidant enzymes. Additionally, GSH-Px plays a crucial role in preventing membrane damage that may result from lipid peroxidation. In this regard, SOD, CAT, and GSH-px activity levels were found to be associated with the ability of the organism to eliminate free radicals from the environment (Stankovic et al., 2020). Related to that, we detected changes in these molecules, where the brain MDA levels in AlCl3 treated group showed a significant increase relative to the control group. Since dopamine and serotonergic signaling are tightly regulated, IMP sems to reverse these abnormalities by preserving serotonergic signaling by directly protecting serotonergic neurons or by maintaining dopamine levels.

**FIGURE 9:**
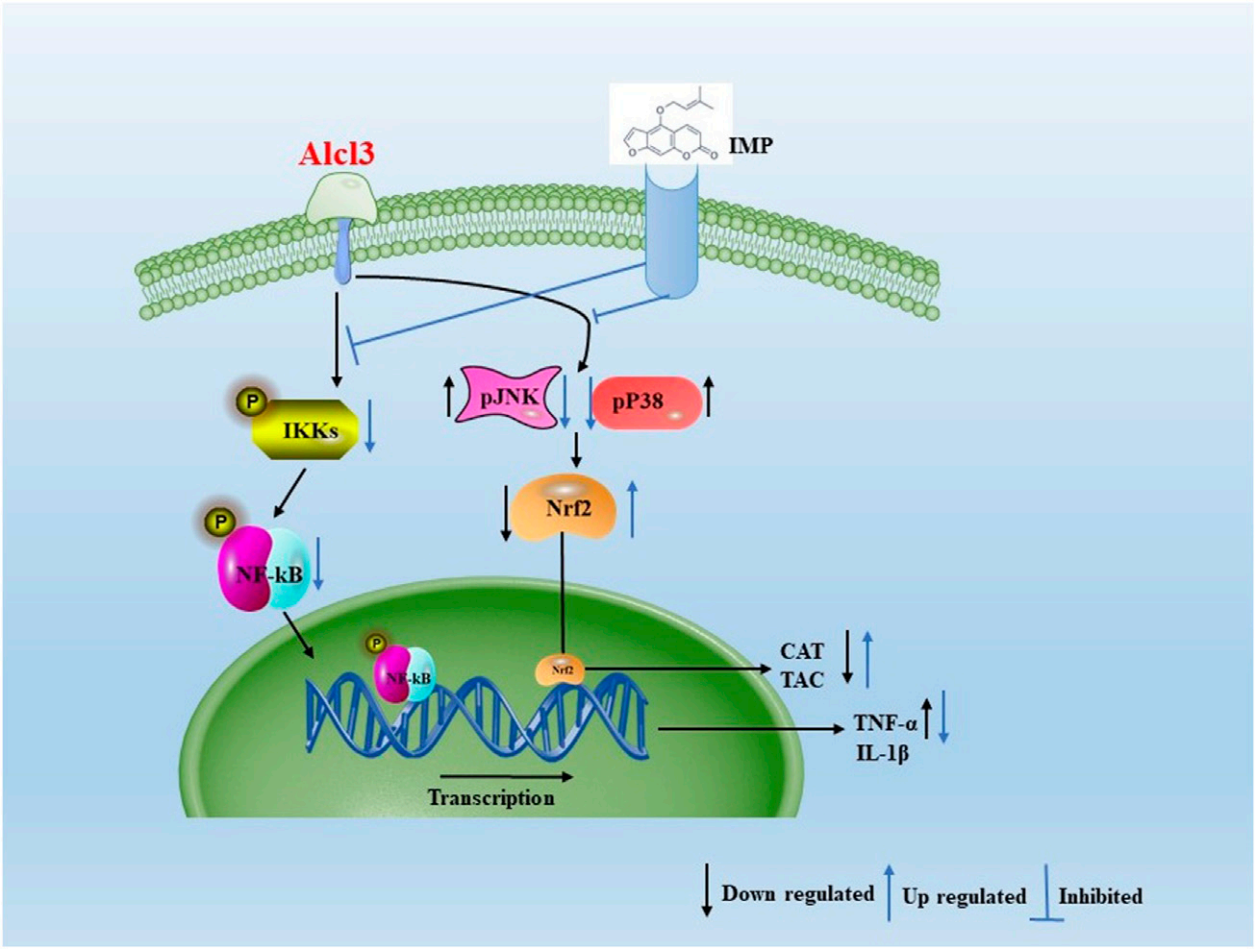
Aproposed scheme to summarizing the obtained results. Mechanistic interaction between signaling molecules is shown. In the present study when mice are treated with AlCl3, inflammation occurs, which increases expression of inflammatory markers such as TNF-α, IL-1β, and NF-kB. TNF-1β, NF-kB, and MAPK activation are attenuated by supplementation with IMP. IMP supplementation attenuates Nrf2 and antioxidant enzymes activation during AlCl3 treatment.

In the current study, IMP increased Nrf2 activation and HO-1, NQO-1, and CAT expression in mouse brain tissue, thereby reducing AlCl3-induced ROS production. AlCl3-induced oxidative stress was attenuated by IMP by activating Nrf2, Nrf2, a critical signaling molecule for ROS detoxification in the brain, is essential for protecting the brain from oxidative stress (Bardallo et al., 2022). In addition, various cellular antioxidants are regulated by Nrf2 (Gureev and Popov, 2019). Phosphorylation of Keap1 causes Nrf2 to migrate to the nucleus when cells it is exposed to neurodegenerative disease-induced oxidative stress (Zhou et al., 2018; Ji et al., 2021; Zong et al., 2021; Mehrbeheshti et al., 2022). In parallel with that, Nrf2 signaling pathway is activated in HT22 cells by antioxidant enzymes such as catalase, NQO-1, and HO-1 (Lone et al., 2018). Thus, the activated Nrf2 as a result of IMP treatment protects the brain from the oxidative damage induced after exposure to AlCl3. In the present study when mice are treated with AlCl3, inflammation occurs, which increases expression of inflammatory markers such as TNF-, IL-1b, and NF-kB. TNF-1b, NF-kB, and MAPK activation are attenuated by supplementation with IMP. IMP supplementation attenuates Nrf2 and antioxidant enzymes activation during AlCl3 treatment. A representative shame to summarize that is shown in Figure 9.

## 4 Conclusion

In the present study, systemic administration of AlCl3 to mice resulted in abnormal behavior, neurotransmitter deficits, and reduced Nrf2 expression in the brain, reflecting oxidative inflammatory stress. Due to its potential antioxidant and anti-inflammatory activities, IMP partially reversed the toxic effects of AlCl3. The results obtained in this work in the AlCl3 model lead us to suggest the IMP as an ideal candidate in diseases that involve processes involving inflammation, dysfunction in synaptic system and neurodegeneration in several areas of the brain and spinal cord.

## Data Availability

The original contributions presented in the study are included in the article/Supplementary Material, further inquiries can be directed to the corresponding authors.
