# Supplementary material for: Isoimperatorin therapeutic effect against aluminum induced neurotoxicity in albino mice
Source: Front Pharmacol. 2023 Apr 18;14:1103940. doi: 10.3389/fphar.2023.1103940 (PMC10172992; doi:10.3389/fphar.2023.1103940)
Supplement: Supplementary file 1 [file Table1.DOCX]

Isoimperatorin therapeutic effect against aluminum induced neurotoxicity in albino mice

**SUPPLEMENTARY TABLES**

**Table S1. Antibody details of the proteins used in immunoblot analysis**

| **Antibody** | **Molecular weight (kDa)** | **Company catalogue** | **Dilution** |
| --- | --- | --- | --- |
| **TNF-α** | 17 | (#PA5-19810) Thermo Fisher Scientific, Inc. (Waltham, MA, USA) | 1:5000 |
| **IL-1β** | 17 | (#PBOIL1BI) Thermo Fisher Scientific, Inc. (Waltham, MA, USA) | 1:5000 |
| **pJNK** | 50 | (#700031) Thermo Fisher Scientific, Inc. (Waltham, MA, USA) | 1:2000 |
| **JNK** | 50 | (# PA5-17634**)** Thermo Fisher Scientific, Inc. (Waltham, MA, USA) | 1:3000 |
| **pP38** | 38 | (#PA5-104910**)** Thermo Fisher Scientific, Inc. (Waltham, MA, USA) |  |
| **P38** | 38 | (#PA5-112391)Thermo Fisher Scientific, Inc. (Waltham, MA, USA) |  |
| **HO-1** | 32 | (#PA5-77833) Thermo Fisher Scientific, Inc. (Waltham, MA, USA) | 1:5000 |
| **NQO-1** | 31 | (#PA5-82294 Thermo Fisher Scientific, Inc. (Waltham, MA, USA) | 1:1000 |
| **p-P65** | 65 | (#MA5-15160), Thermo Fisher Scientific, Inc. (Waltham, MA, USA) | 1:2000 |
| **Nrf2** | 62 | (AB-M-018) MOLEQULE-ON (New Lynn, Auckland, New Zealand) | 1:5000 |
| **secondary antibodies goat anti-rabbit** | - | (AB-M-010) MOLEQULE-ON (New Lynn, Auckland, New Zealand) | 1:10,000 |
| **secondary goat anti-mouse** | - | (AB-M-009) MOLEQULE-ON (New Lynn, Auckland, New Zealand) | 1:10,000 |
| **β-actin** | 43 | (AB-M-003) MOLEQULE-ON (New Lynn, Auckland, New Zealand) | 1:10,000 |
