# Supplementary material for: Isoimperatorin therapeutic effect against aluminum induced neurotoxicity in albino mice
Source: Front Pharmacol. 2023 Apr 18;14:1103940. doi: 10.3389/fphar.2023.1103940 (PMC10172992; doi:10.3389/fphar.2023.1103940)
Supplement: Supplementary file 2 [file Presentation1.PPTX]

## Slide 1
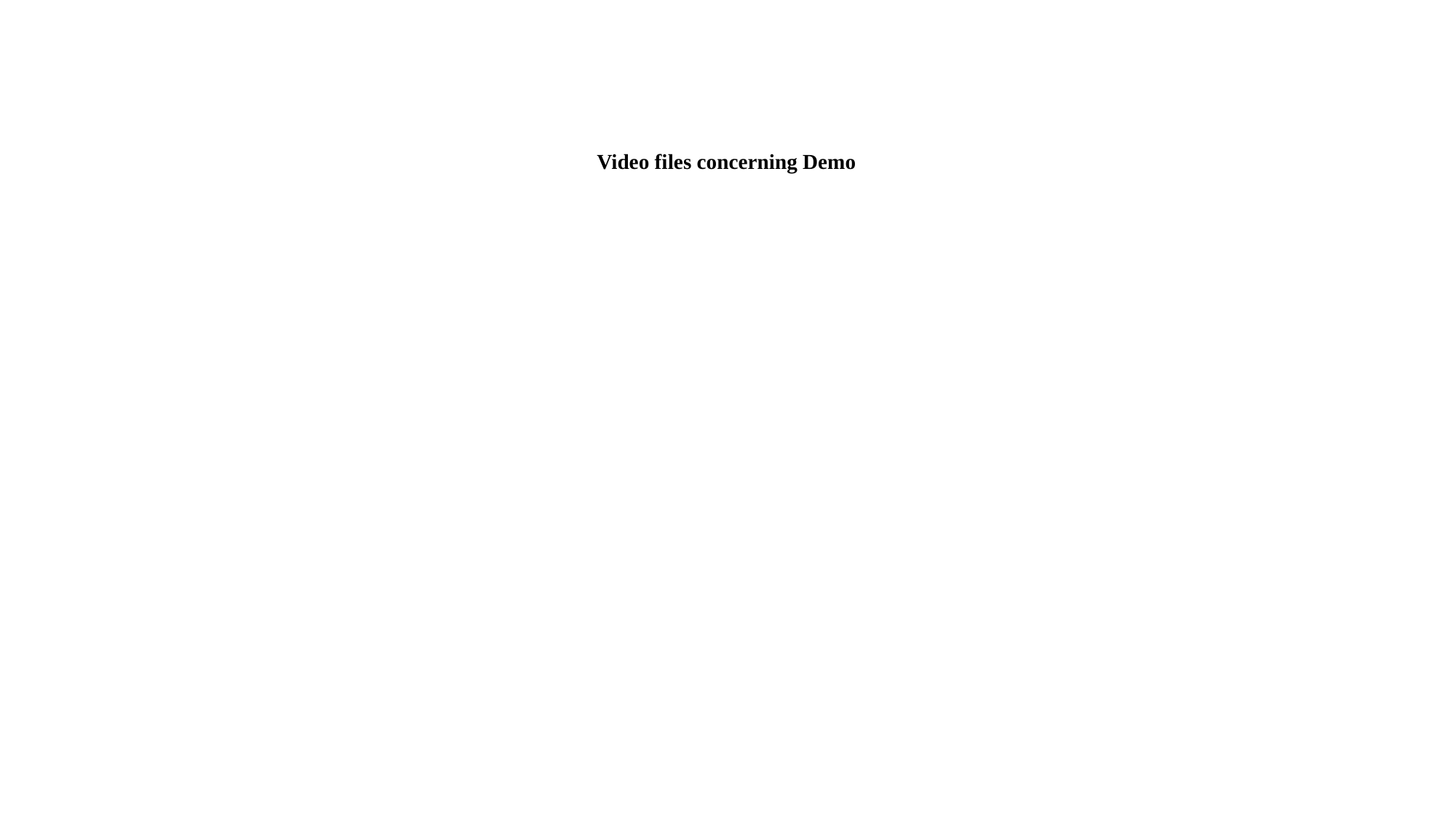

Video files concerning Demo

## Slide 2
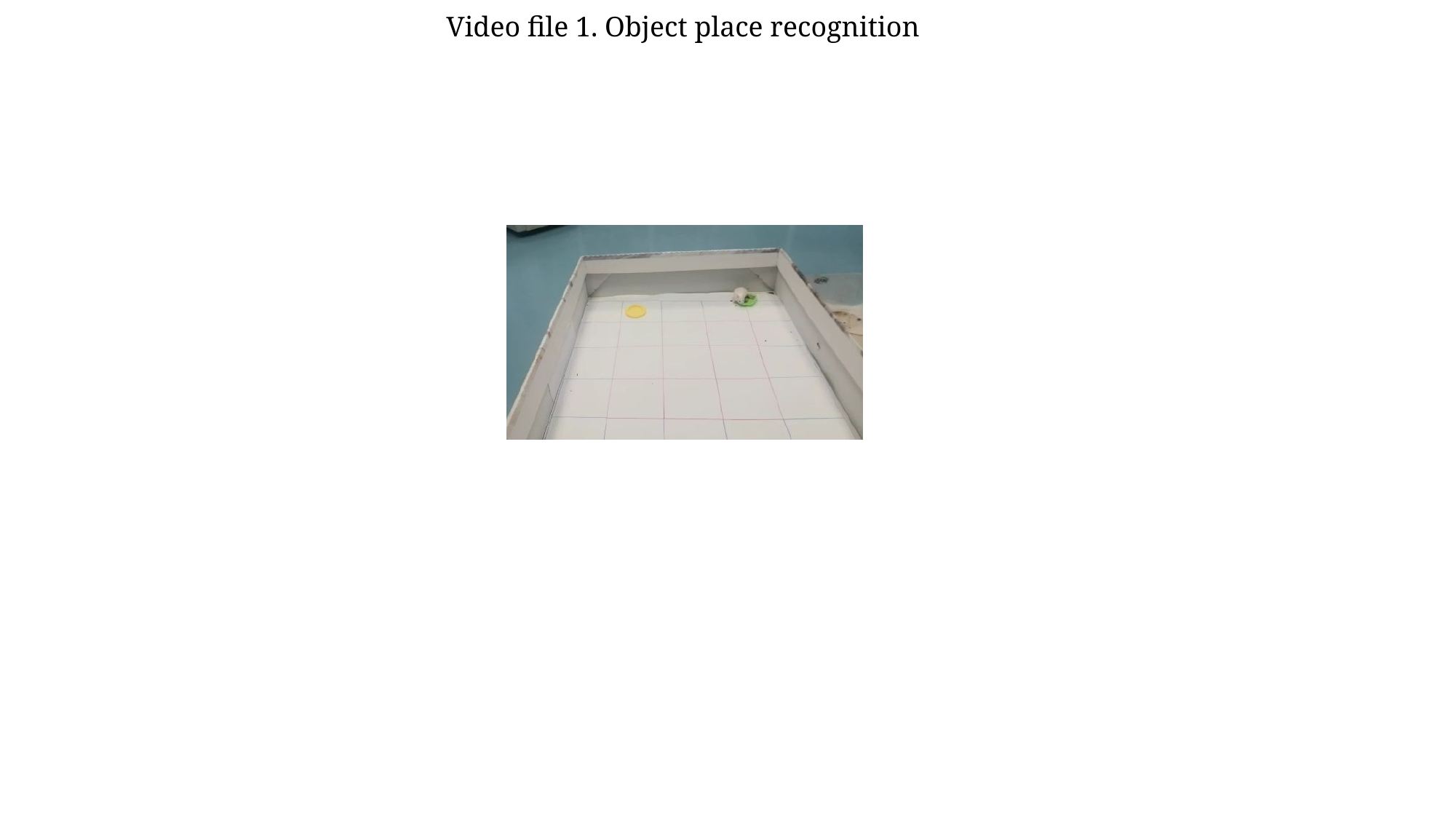

Video file 1. Object place recognition

## Slide 3
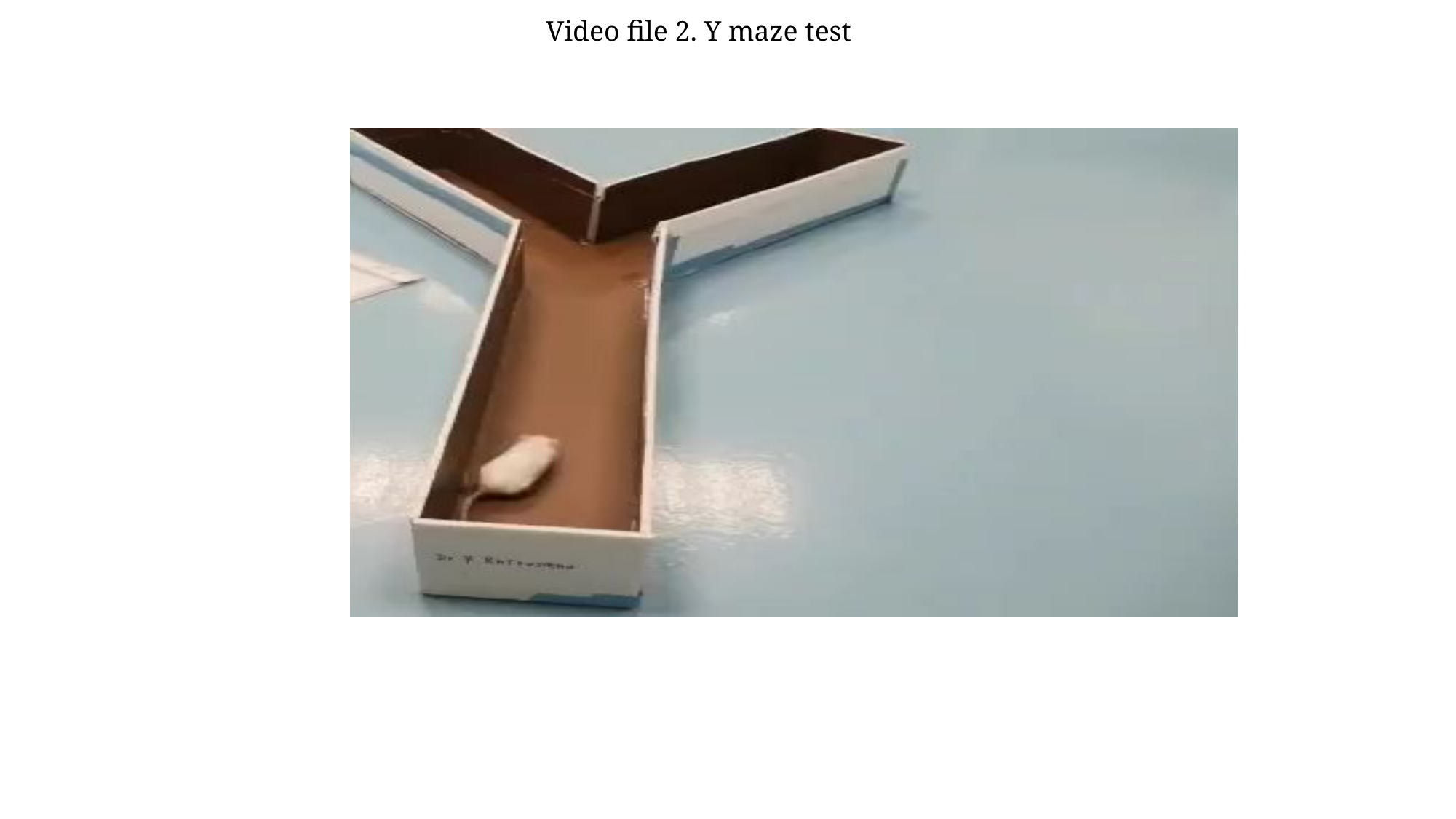

Video file 2. Y maze test
